# Supplementary material for: T1w/T2w Ratio and Cognition in 9-to-11-Year-Old Children
Source: Brain Sci. 2022 May 4;12(5):599. doi: 10.3390/brainsci12050599 (PMC9139105; doi:10.3390/brainsci12050599)

Supplementary Materials

Supplementary Figure S1

Unthresholded, FWE-corrected (across contrasts, vertices, and hemispheres) t-statistical map representing areas in which performance on the Flanker Inhibitory Control and Attention Test was positively associated with T1w/T2w ratio (N=960)

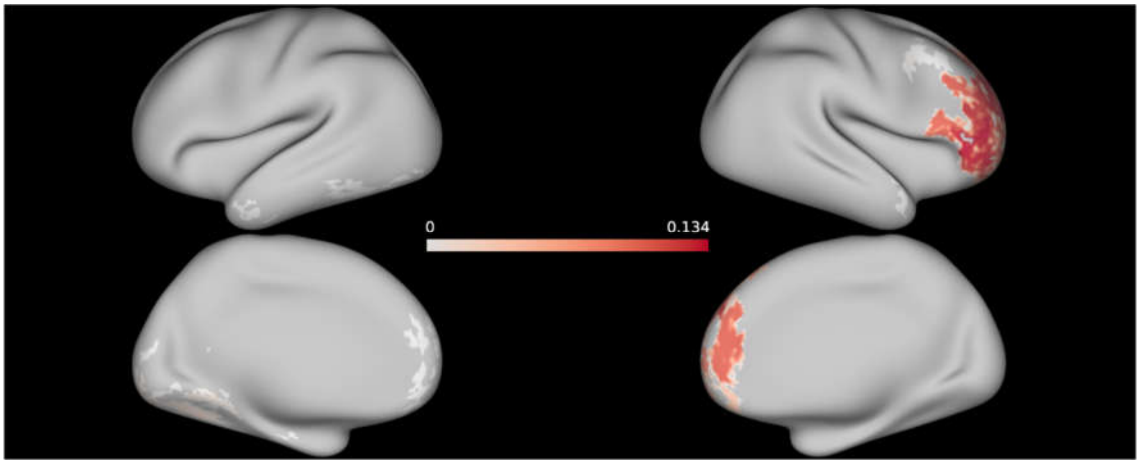

Supplementary Figure S2

Unthresholded, FWE-corrected (across contrasts, vertices, and hemispheres) t-statistical map representing areas in which performance on the Flanker Inhibitory Control and Attention Test was negatively associated with T1w/T2w ratio (N=960)

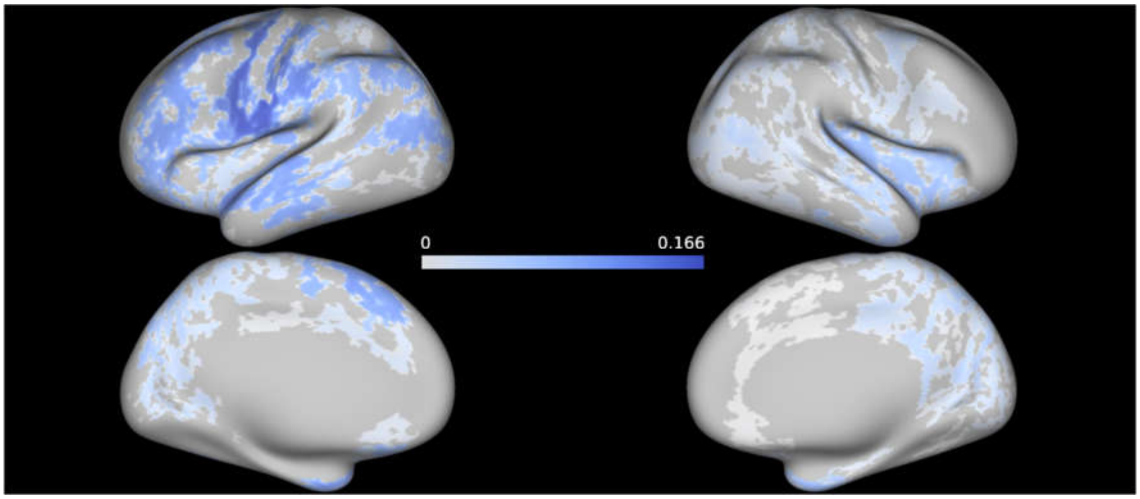

Supplementary Figure S3

Unthresholded, FWE-corrected (across contrasts, vertices, and hemispheres) t-statistical map representing areas in which performance on the List Sorting Working Memory Test was positively associated with T1w/T2w ratio (N=960)

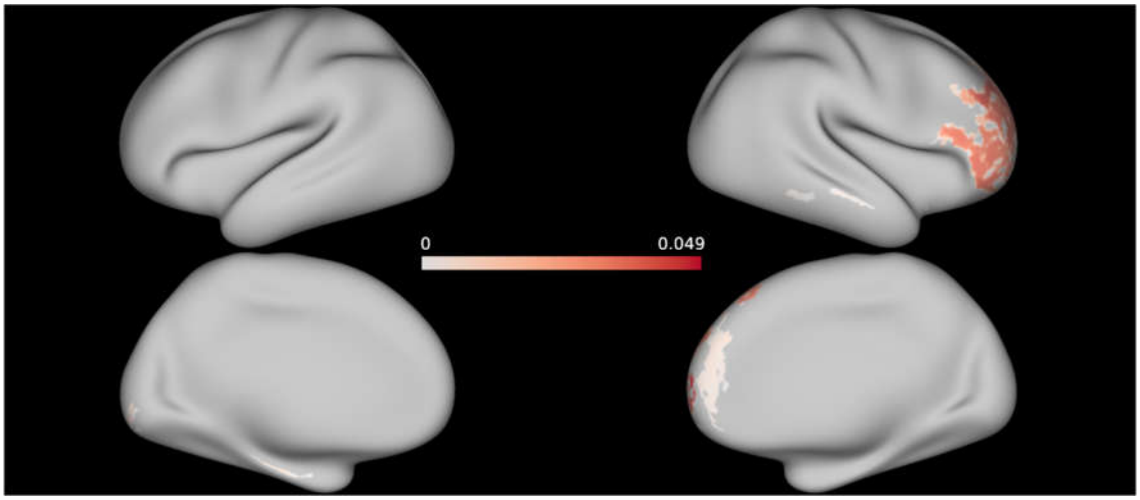

Supplementary Figure S4

Unthresholded, FWE-corrected (across contrasts, vertices, and hemispheres) t-statistical map representing areas in which performance on the List Sorting Working Memory Test was negatively associated with T1w/T2w ratio (N=960)

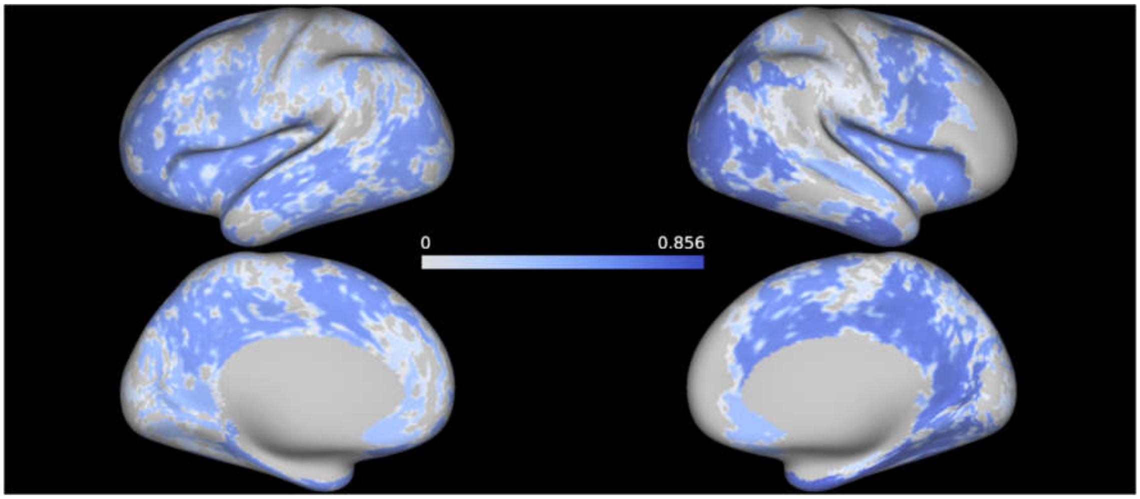

Supplementary Figure S5

Unthresholded, FWE-corrected (across contrasts, vertices, and hemispheres) t-statistical map representing areas in which performance on the Oral Reading Recognition Test was positively associated with T1w/T2w ratio (N=960)

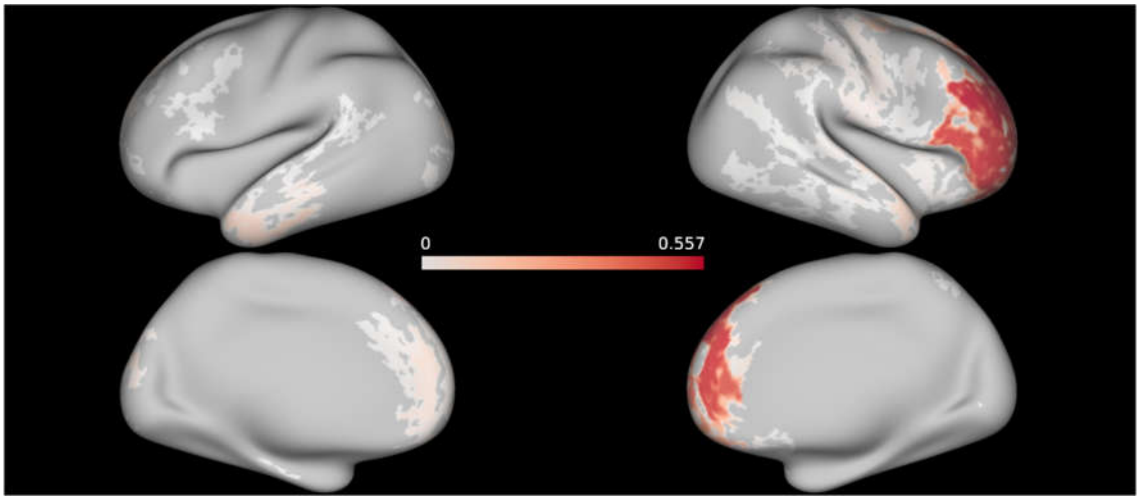

Supplementary Figure S6

Unthresholded, FWE-corrected (across contrasts, vertices, and hemispheres) t-statistical map representing areas in which performance on the Oral Reading Recognition Test was negatively associated with T1w/T2w ratio (N=960)

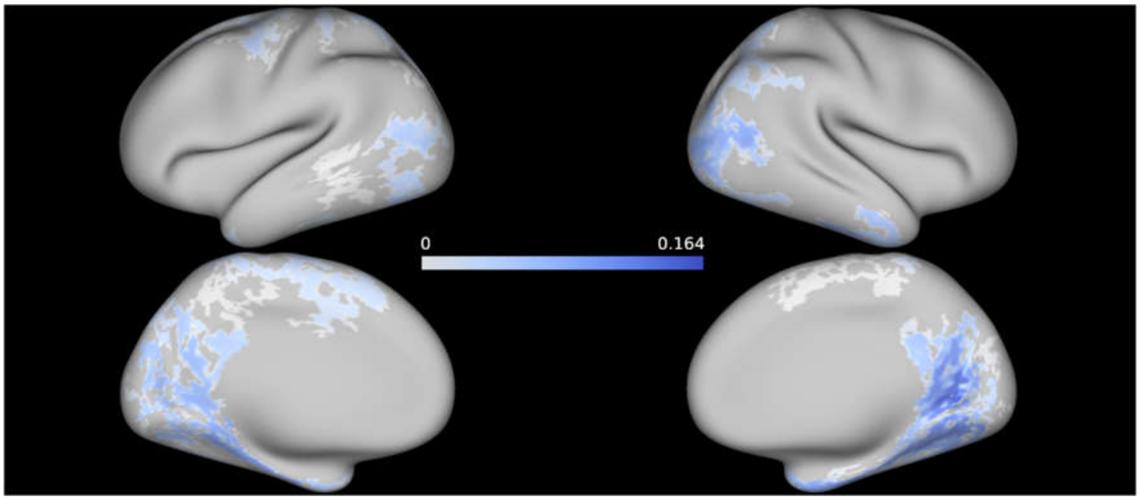

Supplementary Figure S7

Unthresholded, FWE-corrected (across contrasts, vertices, and hemispheres) t-statistical map representing areas in which performance on the Pattern Comparison Processing Speed Test was positively associated with T1w/T2w ratio (N=960)

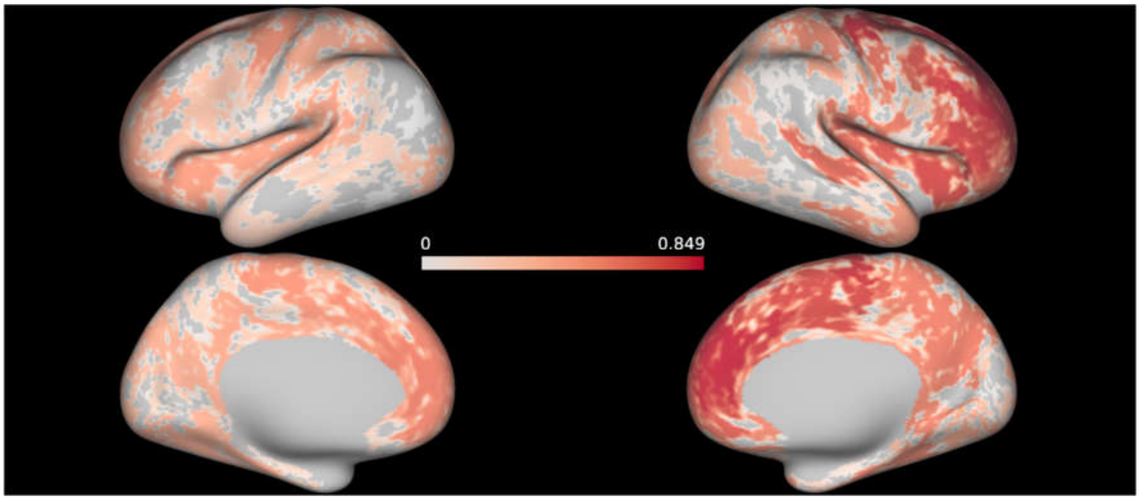

Supplementary Figure S8

Unthresholded, FWE-corrected (across contrasts, vertices, and hemispheres) t-statistical map representing areas in which performance on the Pattern Comparison Processing Speed Test was negatively associated with T1w/T2w ratio (N=960)

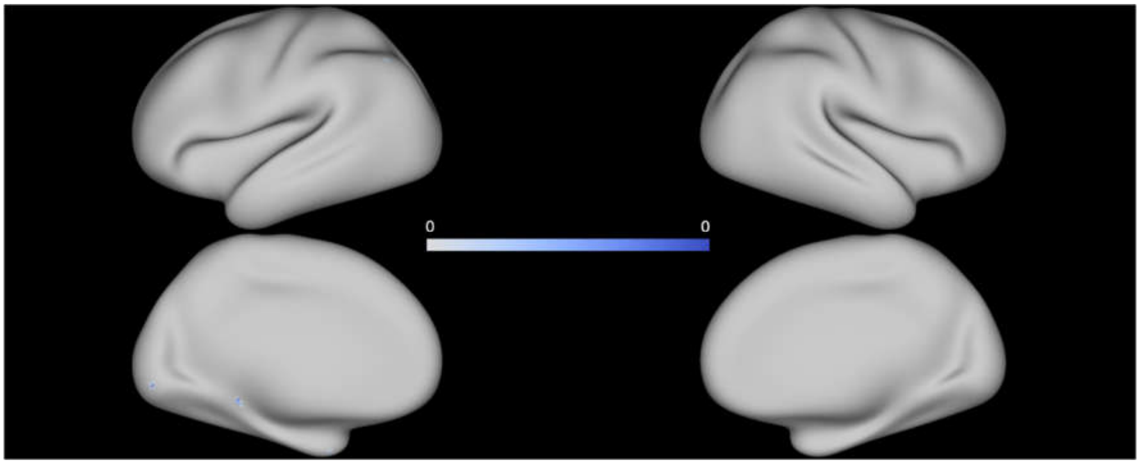

Supplementary Figure S9

Unthresholded, FWE-corrected (across contrasts, vertices, and hemispheres) t-statistical map representing areas in which performance on the Picture Sequence Memory Test was positively associated with T1w/T2w ratio (N=960)

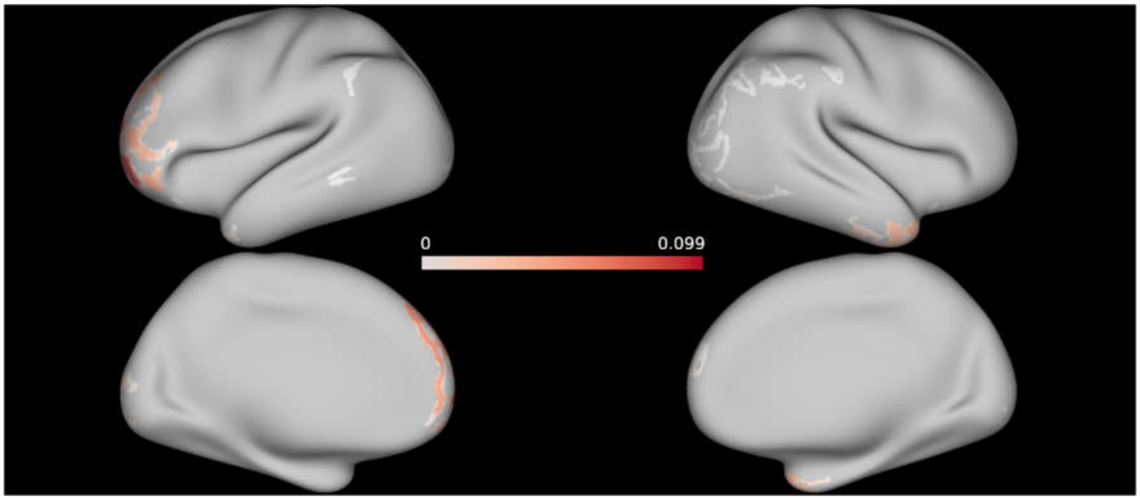

Supplementary Figure S10

Unthresholded, FWE-corrected (across contrasts, vertices, and hemispheres) t-statistical map representing areas in which performance on the Picture Sequence Memory Test was negatively associated with T1w/T2w ratio (N=960)

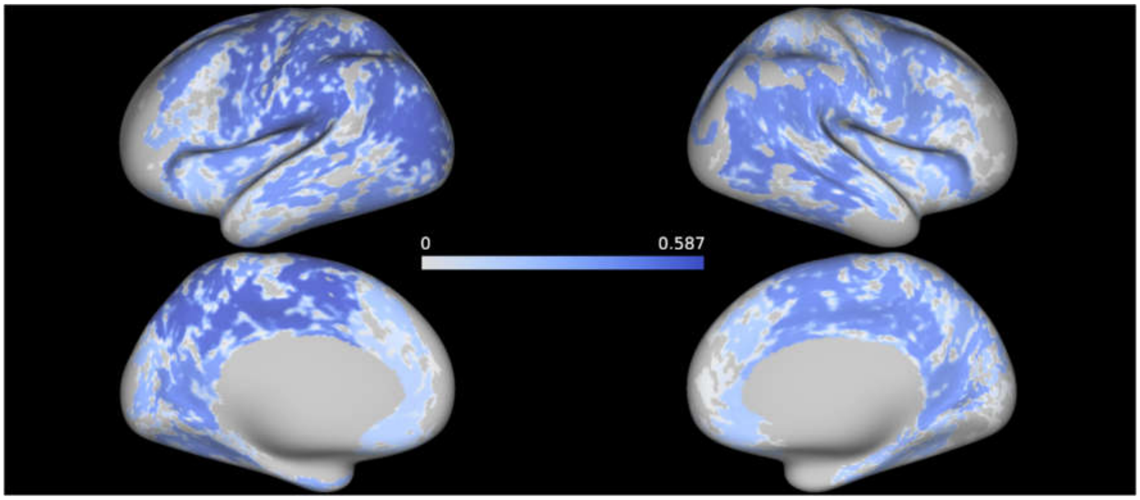

Supplementary Figure S11

Unthresholded, FWE-corrected (across contrasts, vertices, and hemispheres) t-statistical map representing areas in which performance on the Picture Vocabulary Test was positively associated with T1w/T2w ratio (N=960)

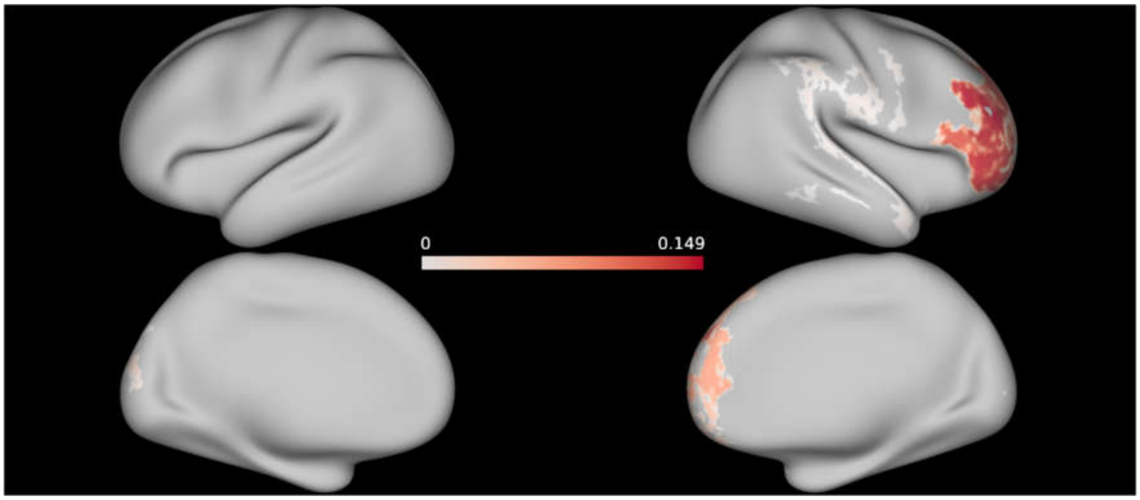

Supplementary Figure S12

Unthresholded, FWE-corrected (across contrasts, vertices, and hemispheres) t-statistical map representing areas in which performance on the Picture Vocabulary Test was negatively associated with T1w/T2w ratio (N=960)

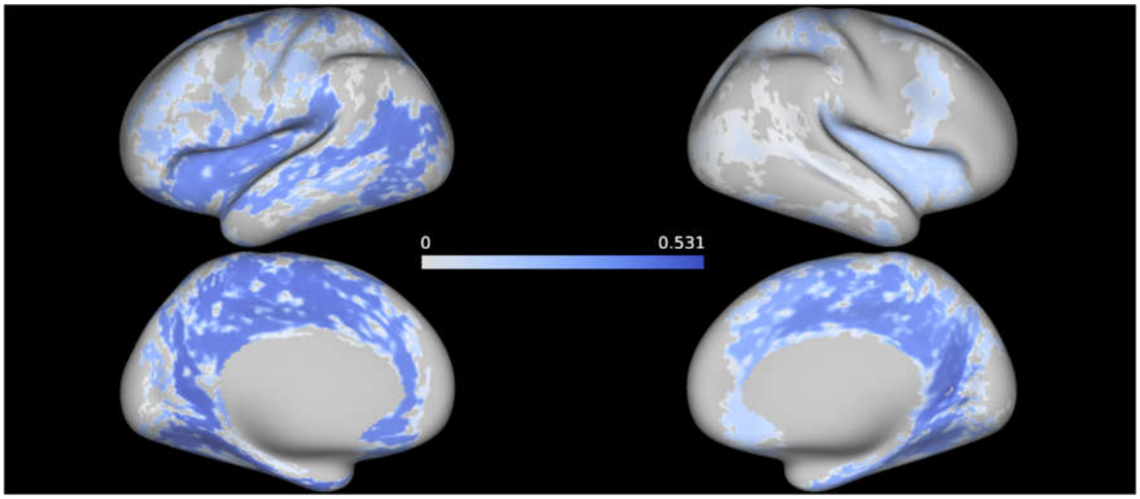

Supplementary Figure S13

Unthresholded, FWE-corrected (across contrasts, vertices, and hemispheres) t-statistical map representing areas in which performance on the Dimensional Change Card Sort Test was positively associated with T1w/T2w ratio (N=960)

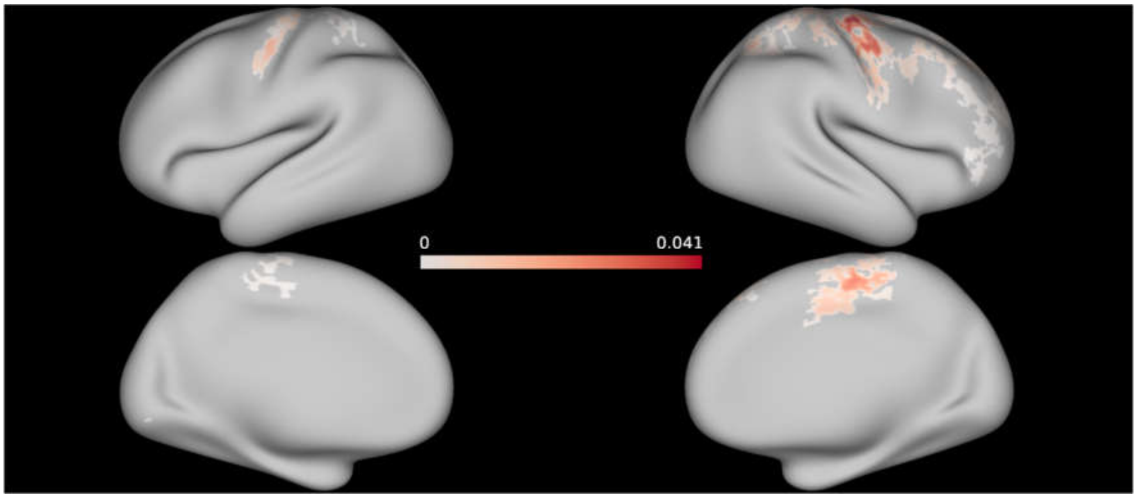

Supplementary Figure S14

Unthresholded, FWE-corrected (across contrasts, vertices, and hemispheres) t-statistical map representing areas in which performance on the Dimensional Change Card Sort Test was negatively associated with T1w/T2w ratio (N=960)

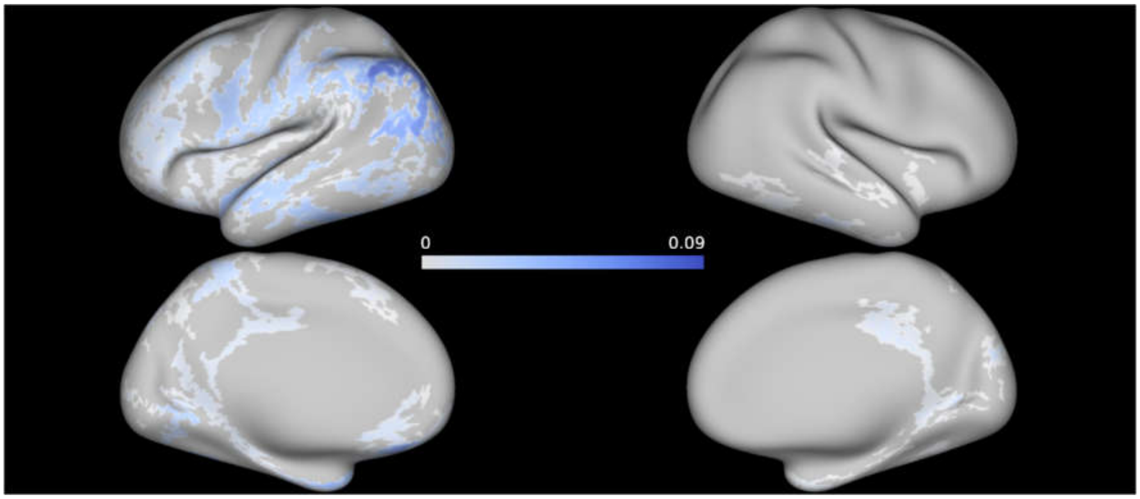

Supplementary Figure S15

Distribution of uncorrected scores on Flanker Inhibitory Control and Attention Test. Participants located more than 1.5 times the interquartile range below the first or over the third quartile are marked as outliers

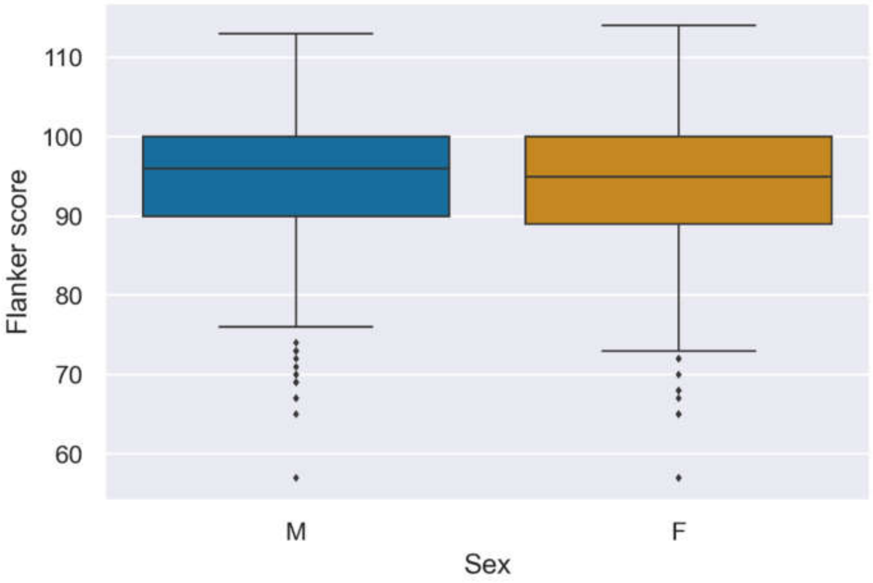

Supplementary Figure S16

Distribution of uncorrected scores on List Sorting Working Memory Test. Participants located more than 1.5 times the interquartile range below the first or over the third quartile are marked as outliers

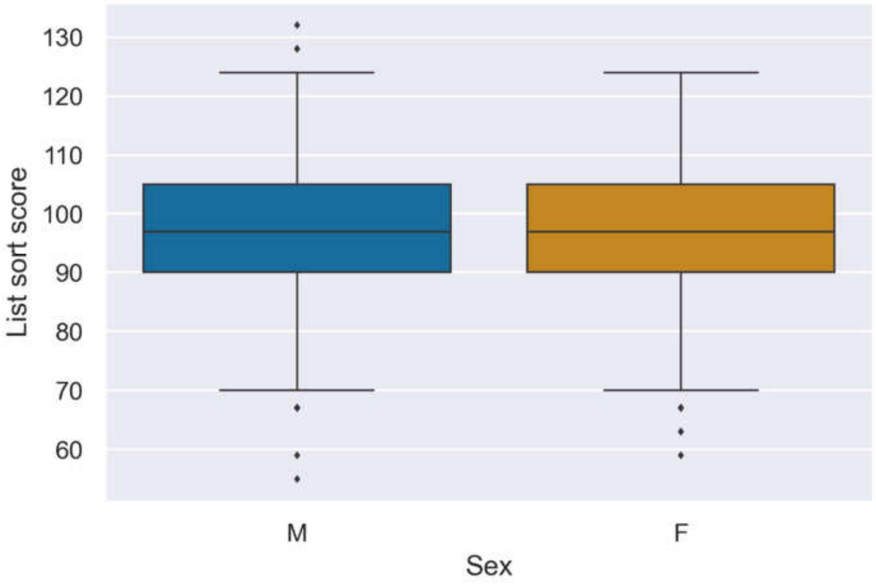

Supplementary Figure S17

Distribution of uncorrected scores on Oral Reading Recognition Test. Participants located more than 1.5 times the interquartile range below the first or over the third quartile are marked as outliers

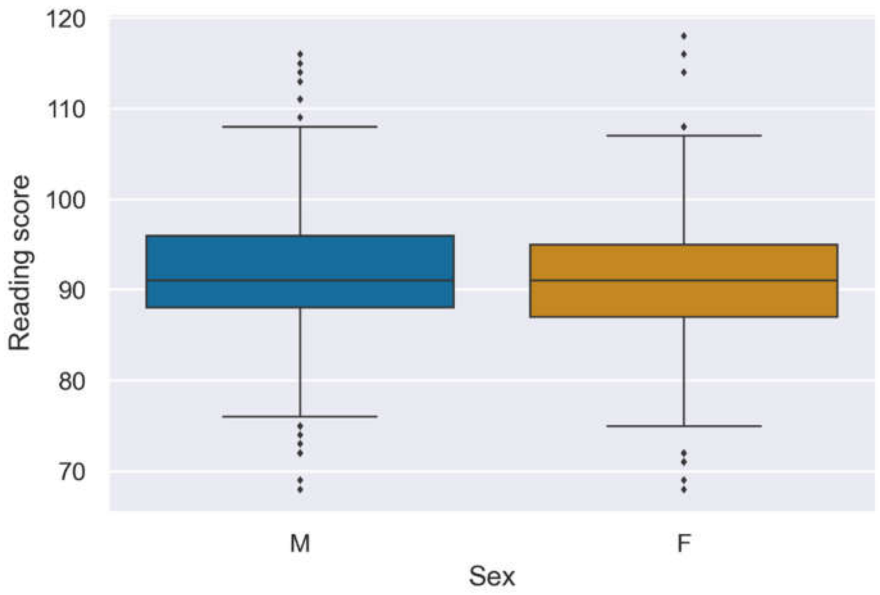

Supplementary Figure S18

Distribution of uncorrected scores on Pattern Comparison Processing Speed Test. Participants located more than 1.5 times the interquartile range below the first or over the third quartile are marked as outliers

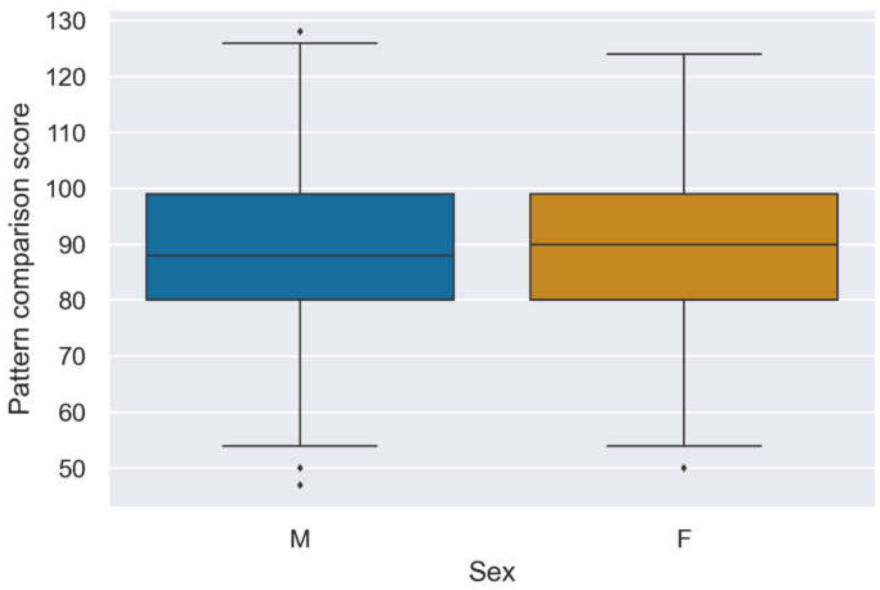

Supplementary Figure S19

Distribution of uncorrected scores on Picture Sequence Memory Test. Participants located more than 1.5 times the interquartile range below the first or over the third quartile are marked as outliers

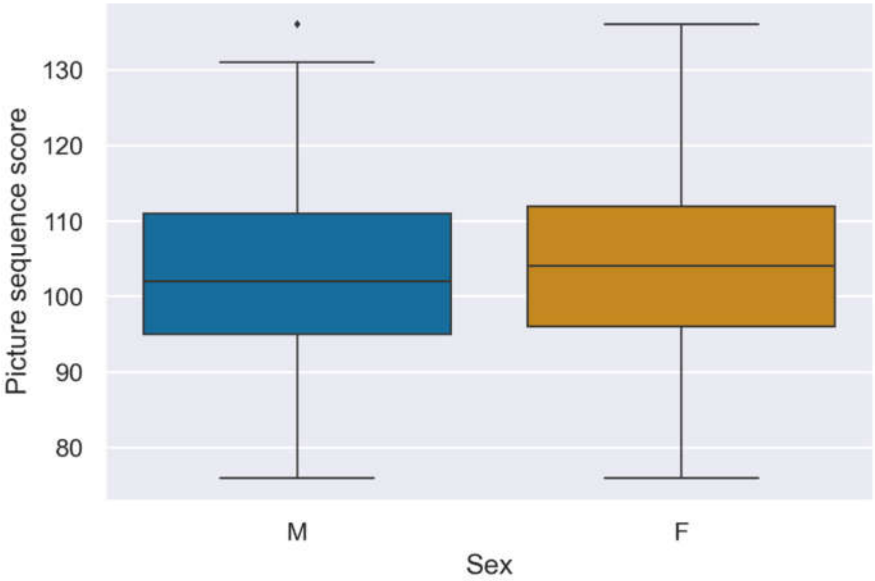

Supplementary Figure S20

Distribution of uncorrected scores on Picture Vocabulary Test. Participants located more than 1.5 times the interquartile range below the first or over the third quartile are marked as outliers

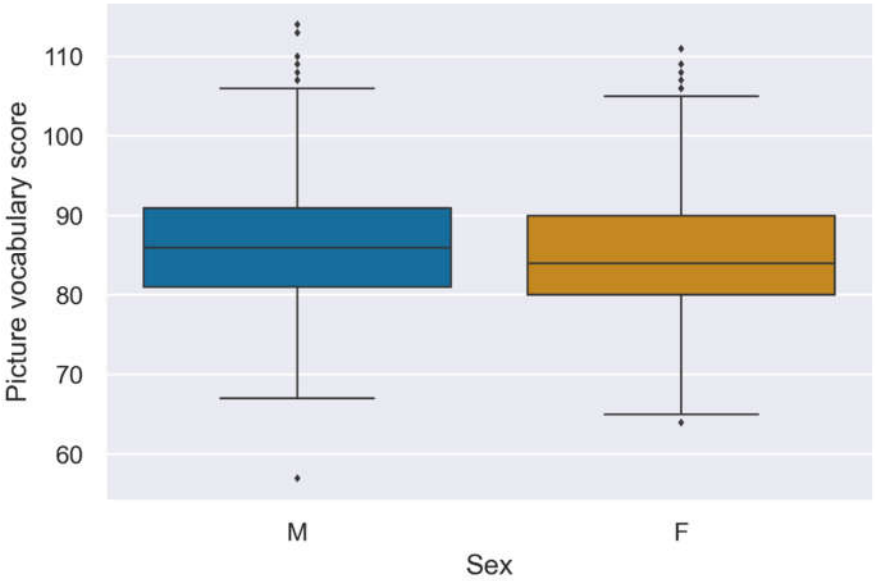

Supplementary Figure S21

Distribution of uncorrected scores on Dimensional Change Card Sort Test. Participants located more than 1.5 times the interquartile range below the first or over the third quartile are marked as outliers

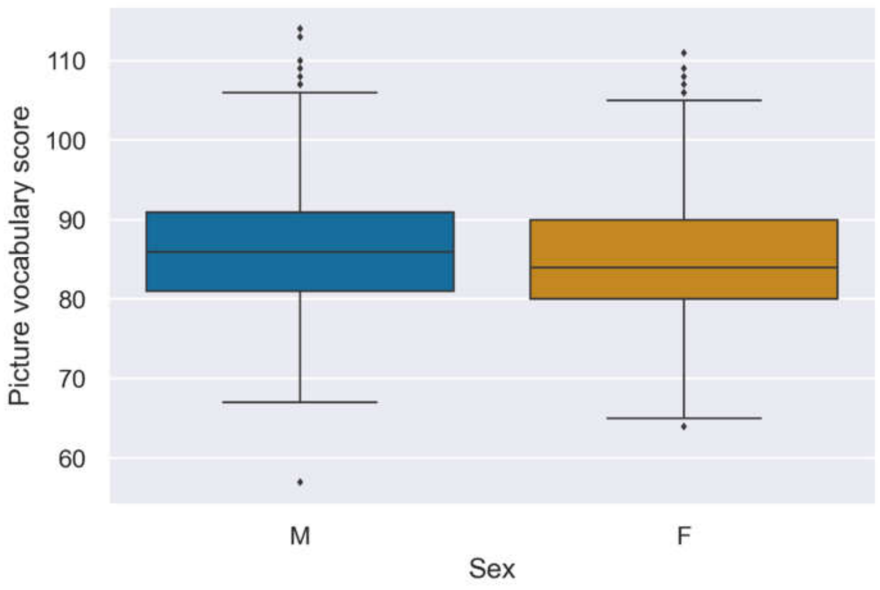

***Supplementary Figure S22** (a-c)*

Example maps representing individual cortical myelination patterns for three age-matched (127 months) participants. Individual maps are projected onto the sample's average inflated surface. Warm colors indicate higher degree and cold colors indicate lower degree of myelination

(a)

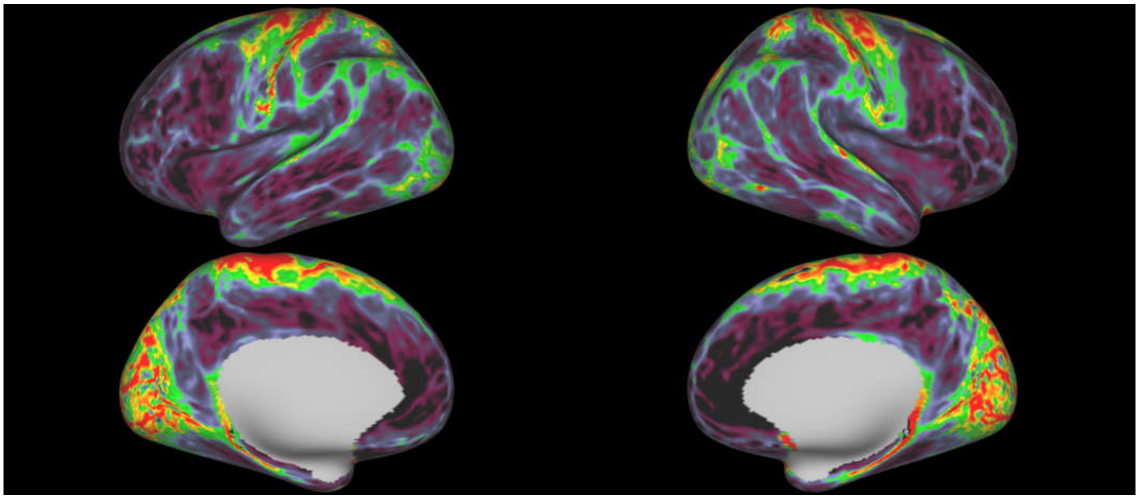

(b)

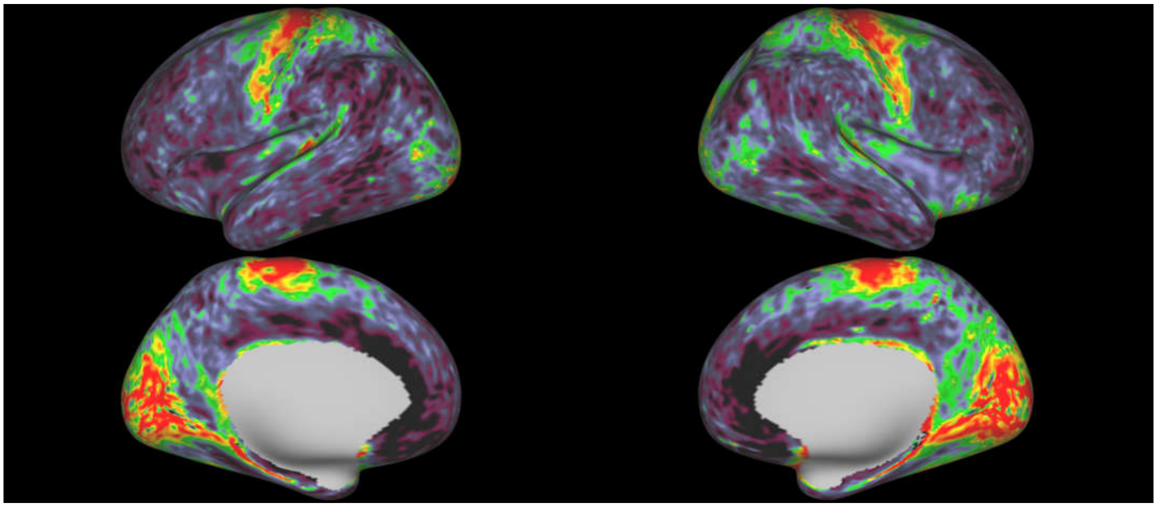

(c)

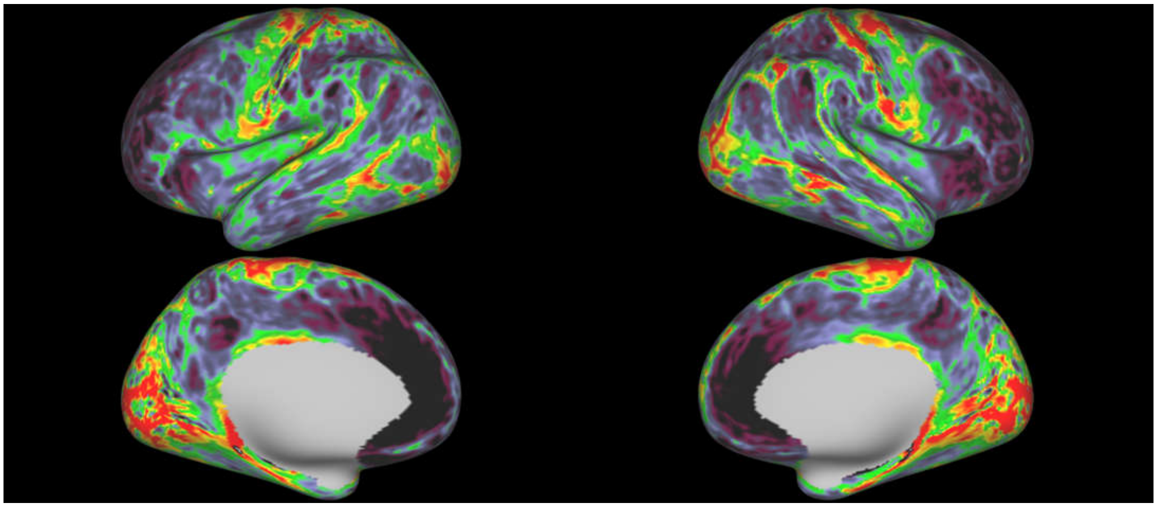

Supplementary Figure S23

Unthresholded, FWE-corrected (across contrasts, vertices, and hemispheres) t-statistical map representing areas in which age was positively associated with T1w/T2w ratio (N=953)

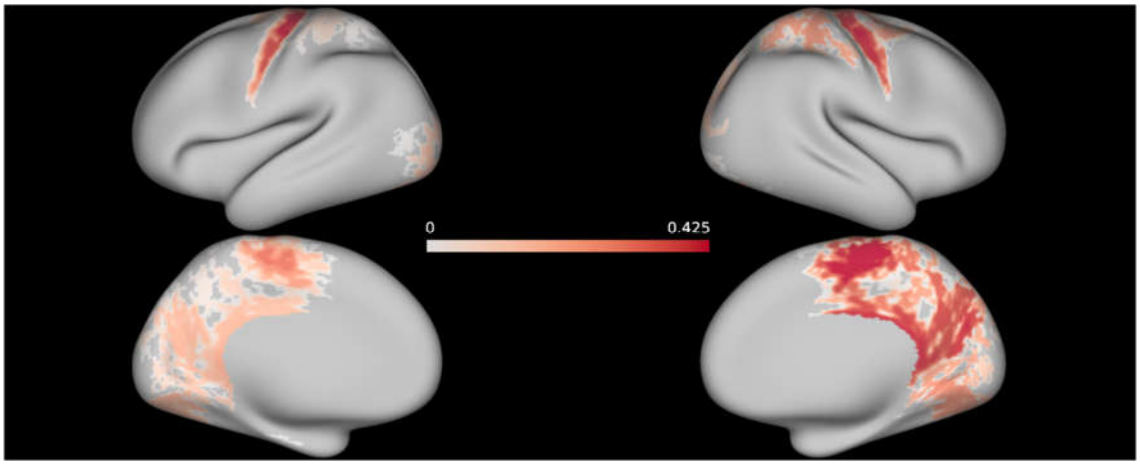

Supplementary Figure S24

Unthresholded, FWE-corrected (across contrasts, vertices, and hemispheres) t-statistical map representing areas in which age was negatively associated with T1w/T2w ratio (N=953)

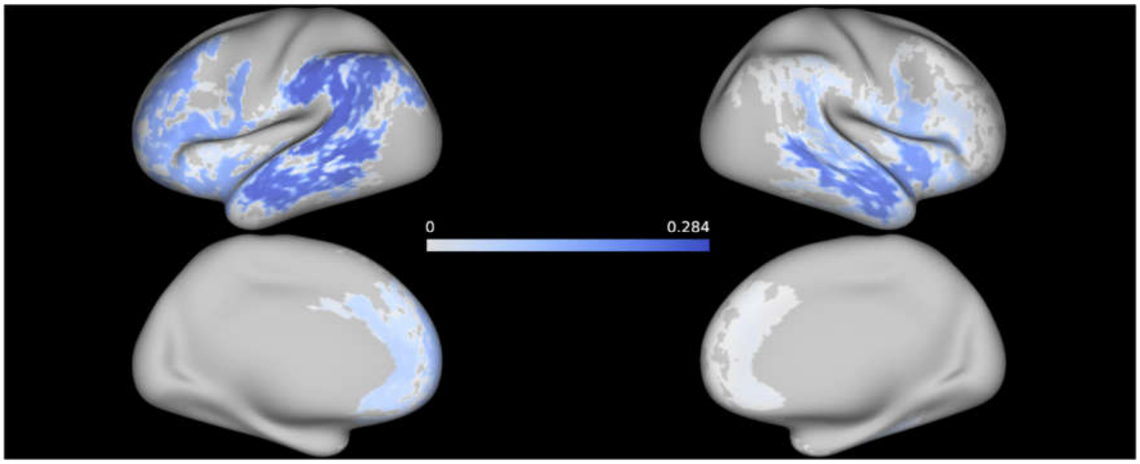

Supplementary Figure S25

Unthresholded, FWE-corrected (across contrasts, vertices, and hemispheres) t-statistical map representing areas in which sex was positively associated with T1w/T2w ratio (N=953)

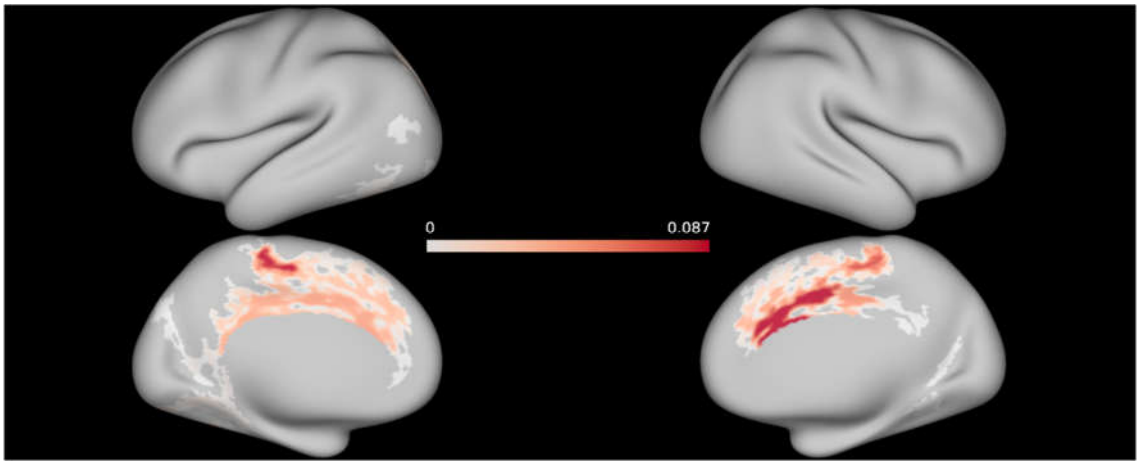

Supplementary Figure S26

Unthresholded, FWE-corrected (across contrasts, vertices, and hemispheres) t-statistical map representing areas in which sex was negatively associated with T1w/T2w ratio (N=953)

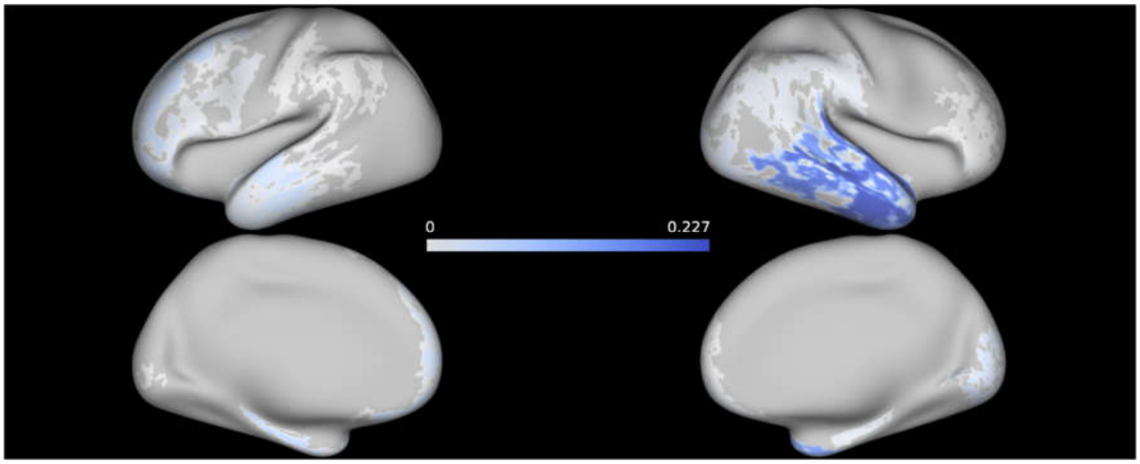

Supplementary Figure S27

Unthresholded, FWE-corrected (across contrasts, vertices, and hemispheres) t-statistical map representing areas in which SES was positively associated with T1w/T2w ratio (N=953)

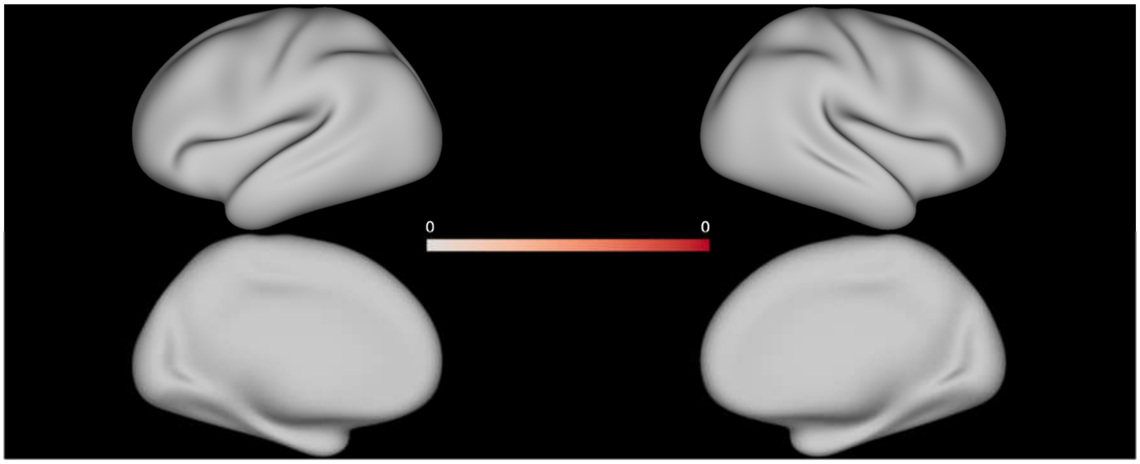

Supplementary Figure S28

Unthresholded, FWE-corrected (across contrasts, vertices, and hemispheres) t-statistical map representing areas in which SES was negatively associated with T1w/T2w ratio (N=953)

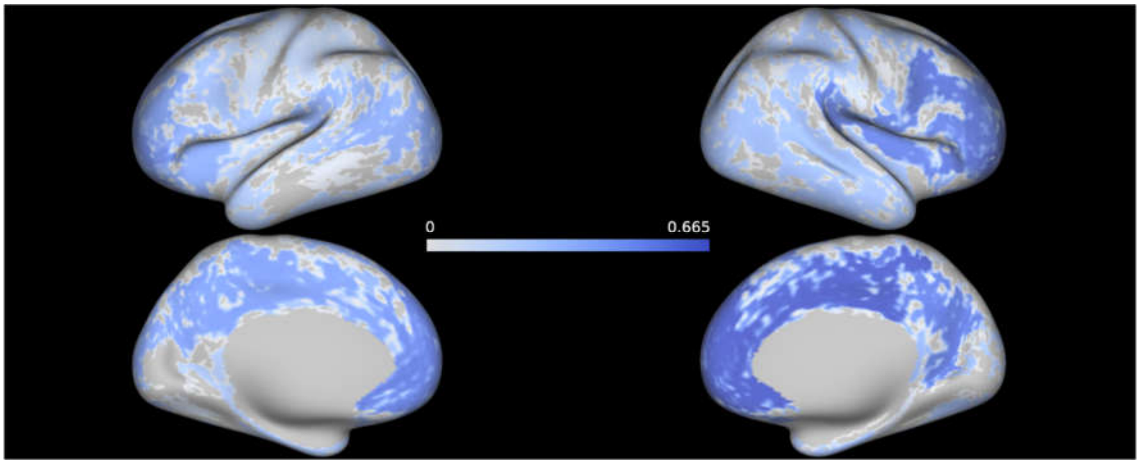

Supplementary Figure S29

Unthresholded, FWE-corrected (across contrasts, vertices, and hemispheres) t-statistical map representing areas in which performance on the Flanker Inhibitory Control and Attention Test was positively associated with T1w/T2w ratio (N=953) when scanner site, age, sex, and SES were included as covariates of no interest.

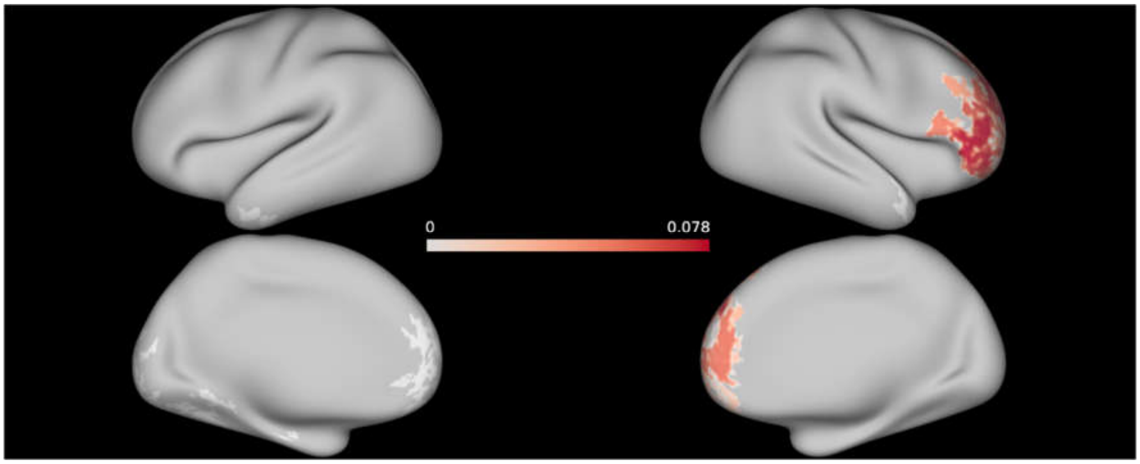

Supplementary Figure S30

Unthresholded, FWE-corrected (across contrasts, vertices, and hemispheres) t-statistical map representing areas in which performance on the Flanker Inhibitory Control and Attention Test was negatively associated with T1w/T2w ratio (N=953) when scanner site, age, sex, and SES were included as covariates of no interest.

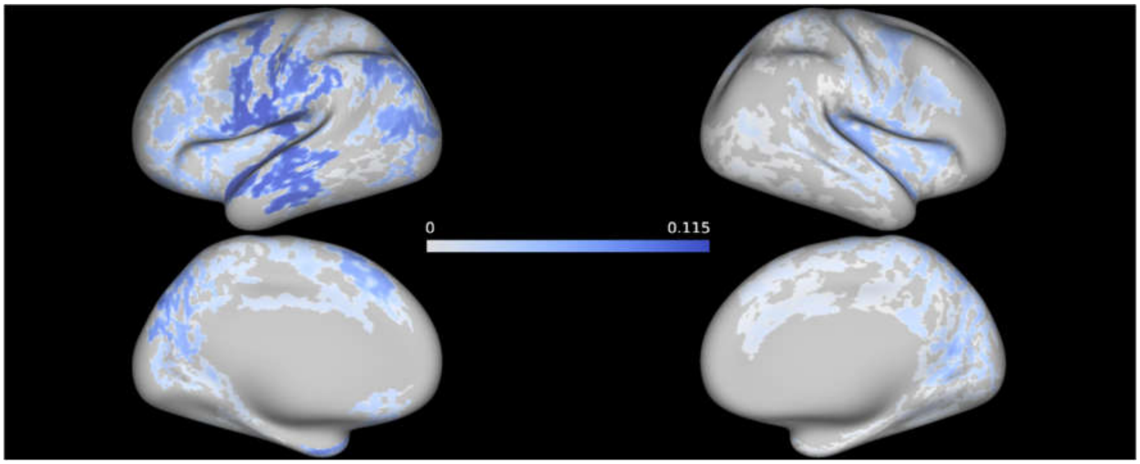

Supplementary Figure S31

Unthresholded, FWE-corrected (across contrasts, vertices, and hemispheres) t-statistical map representing areas in which performance on the List Sorting Working Memory Test was positively associated with T1w/T2w ratio (N=953) when scanner site, age, sex, and SES were included as covariates of no interest.

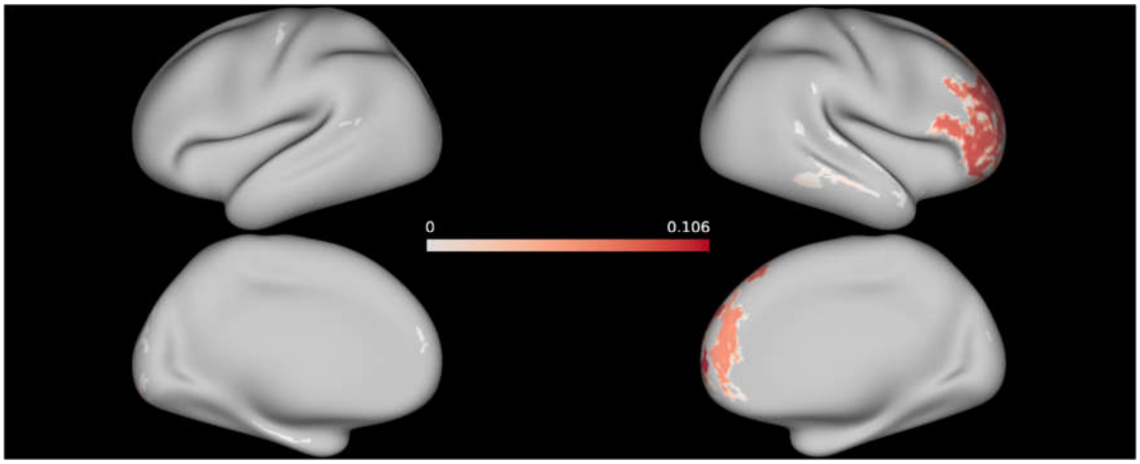

Supplementary Figure S32

Unthresholded, FWE-corrected (across contrasts, vertices, and hemispheres) t-statistical map representing areas in which performance on the List Sorting Working Memory Test was negatively associated with T1w/T2w ratio (N=953) when scanner site, age, sex, and SES were included as covariates of no interest.

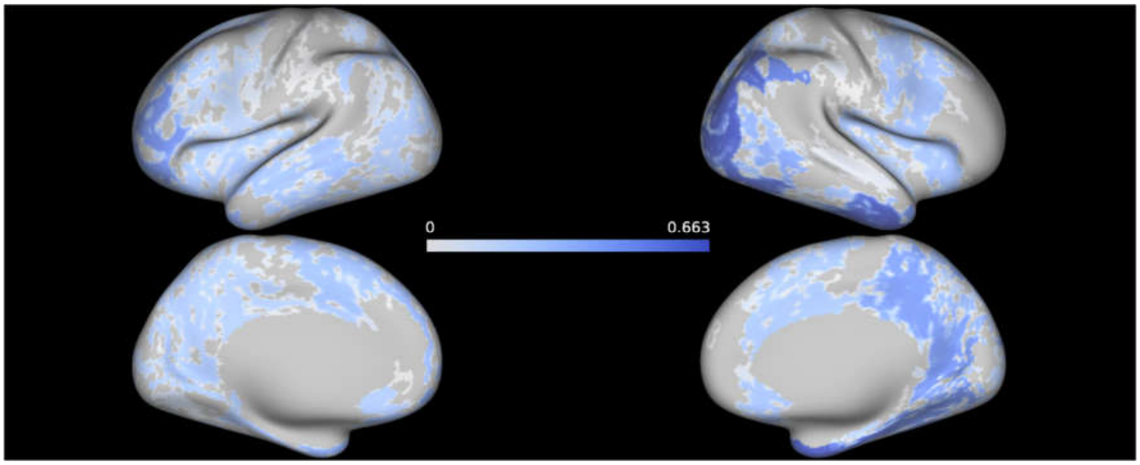

Supplementary Figure S32

Unthresholded, FWE-corrected (across contrasts, vertices, and hemispheres) t-statistical map representing areas in which performance on the Oral Reading Recognition Test was positively associated with T1w/T2w ratio (N=953) when scanner site, age, sex, and SES were included as covariates of no interest.

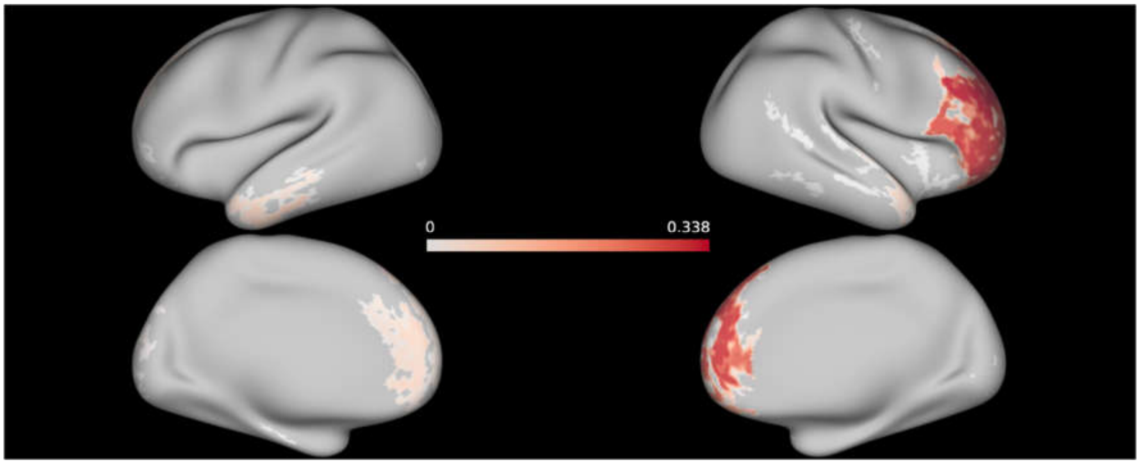

Supplementary Figure S33

Unthresholded, FWE-corrected (across contrasts, vertices, and hemispheres) t-statistical map representing areas in which performance on the List Sorting Working Memory Test was negatively associated with T1w/T2w ratio (N=953) when scanner site, age, sex, and SES were included as covariates of no interest.

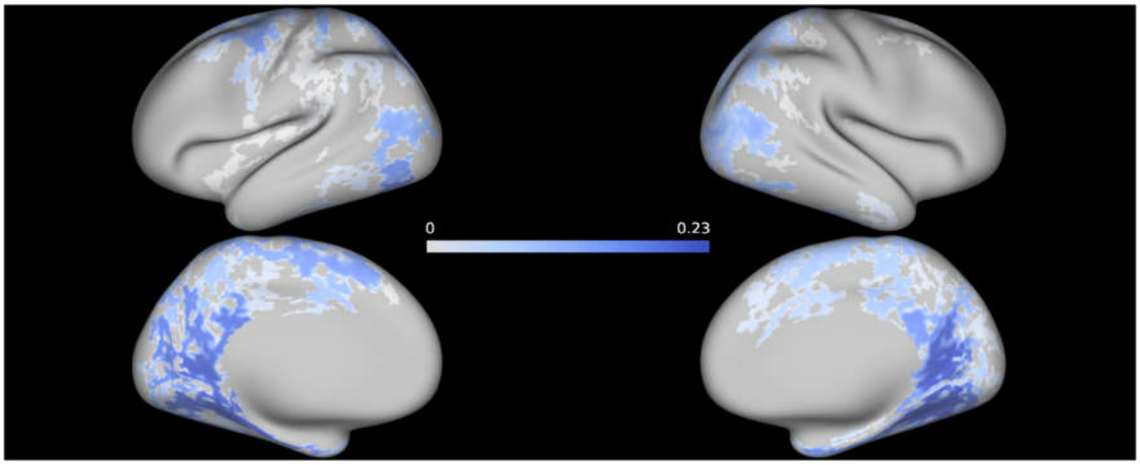

Supplementary Figure S34

Unthresholded, FWE-corrected (across contrasts, vertices, and hemispheres) t-statistical map representing areas in which performance on the Pattern Comparison Processing Speed Test was positively associated with T1w/T2w ratio (N=953) when scanner site, age, sex, and SES were included as covariates of no interest.

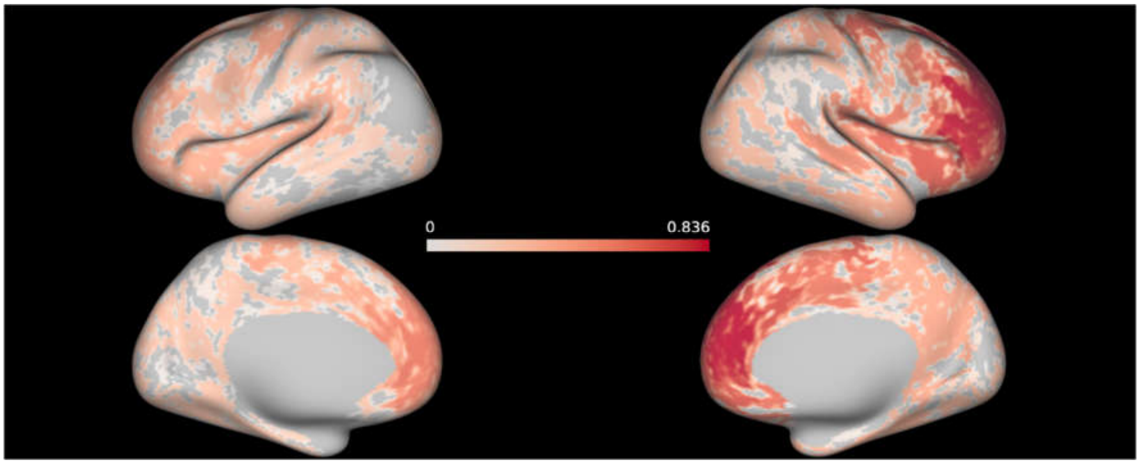

Supplementary Figure S35

Unthresholded, FWE-corrected (across contrasts, vertices, and hemispheres) t-statistical map representing areas in which performance on the Pattern Comparison Processing Speed Test was negatively associated with T1w/T2w ratio (N=953) when scanner site, age, sex, and SES were included as covariates of no interest.

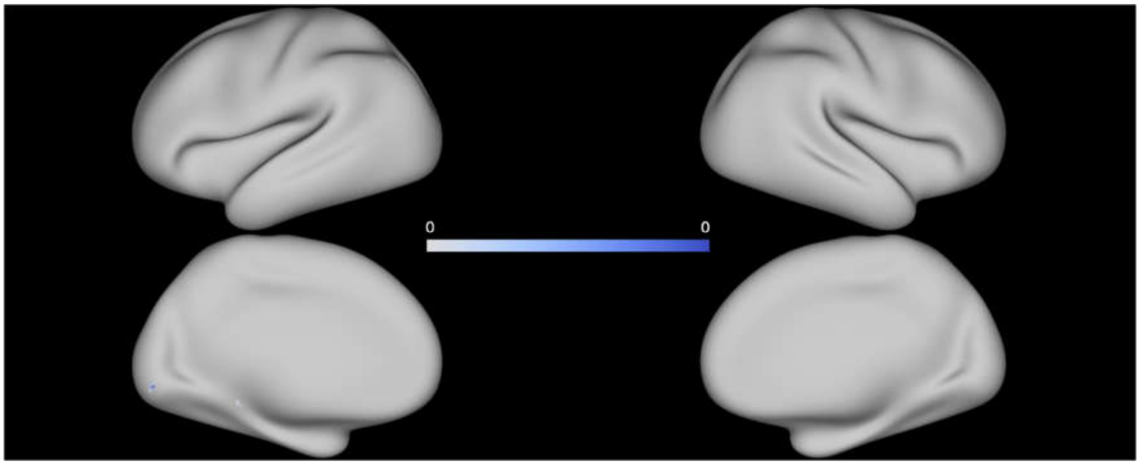

Supplementary Figure S36

Unthresholded, FWE-corrected (across contrasts, vertices, and hemispheres) t-statistical map representing areas in which performance on the Picture Sequence Memory Test was positively associated with T1w/T2w ratio (N=953) when scanner site, age, sex, and SES were included as covariates of no interest.

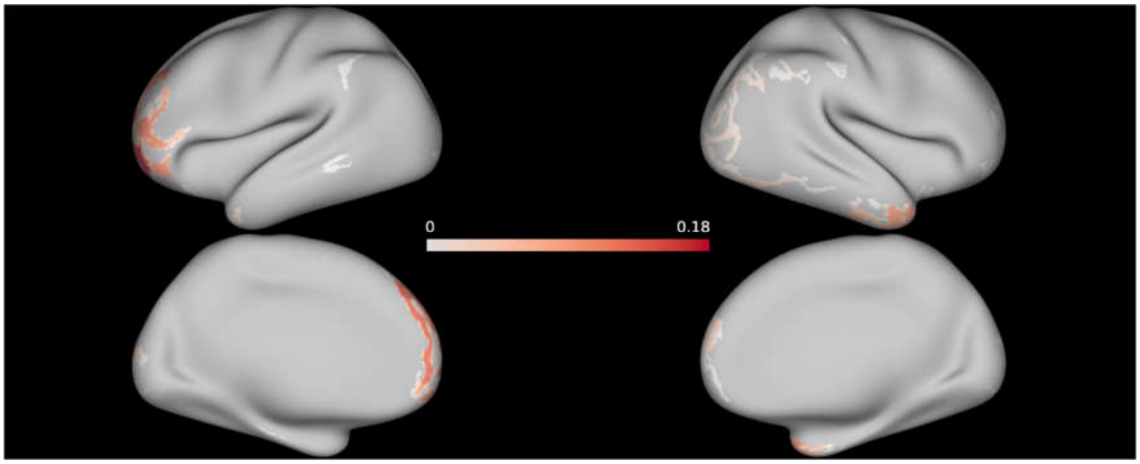

Supplementary Figure S37

Unthresholded, FWE-corrected (across contrasts, vertices, and hemispheres) t-statistical map representing areas in which performance on the Picture Sequence Memory Test was negatively associated with T1w/T2w ratio (N=953) when scanner site, age, sex, and SES were included as covariates of no interest.

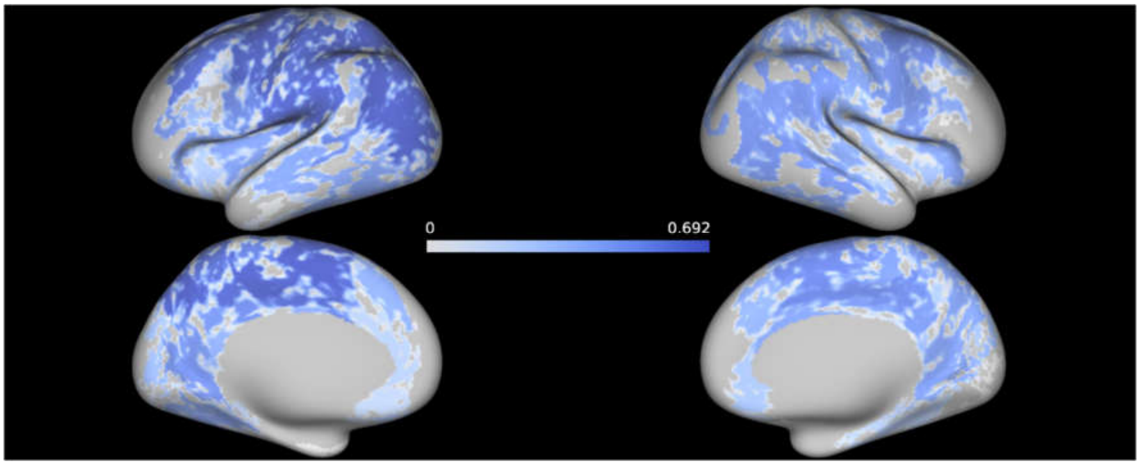

Supplementary Figure S38

Unthresholded, FWE-corrected (across contrasts, vertices, and hemispheres) t-statistical map representing areas in which performance on the Picture Vocabulary Test was positively associated with T1w/T2w ratio (N=953) when scanner site, age, sex, and SES were included as covariates of no interest.

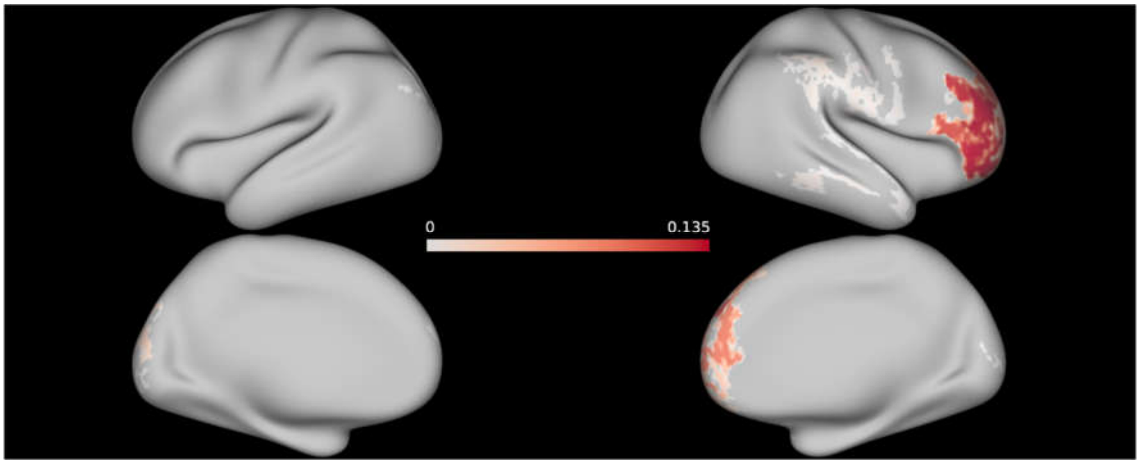

Supplementary Figure S34

Unthresholded, FWE-corrected (across contrasts, vertices, and hemispheres) t-statistical map representing areas in which performance on the Picture Vocabulary Test was positively associated with T1w/T2w ratio (N=953) when scanner site, age, sex, and SES were included as covariates of no interest.

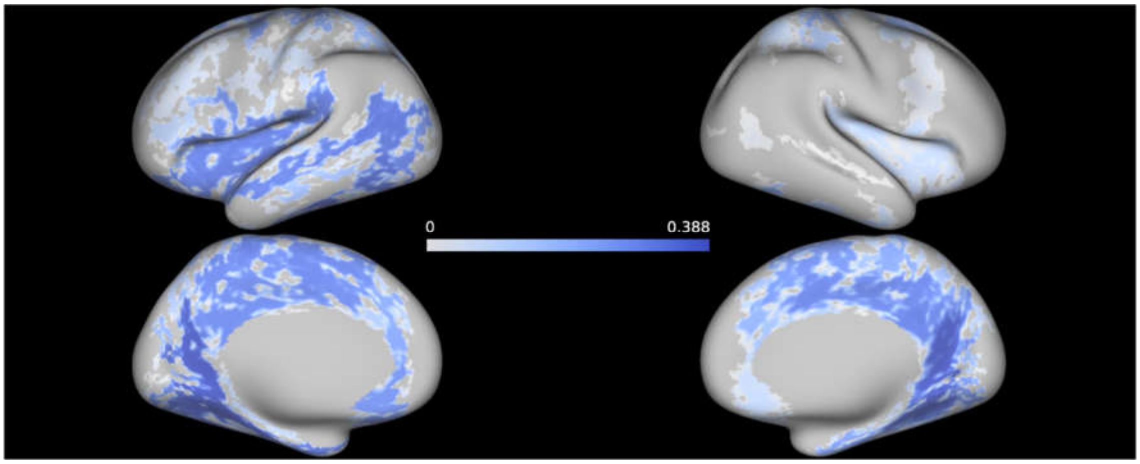

Supplementary Figure S40

Unthresholded, FWE-corrected (across contrasts, vertices, and hemispheres) t-statistical map representing areas in which performance on the Dimensional Change Card Sort Test was positively associated with T1w/T2w ratio (N=953) when scanner site, age, sex, and SES were included as covariates of no interest.

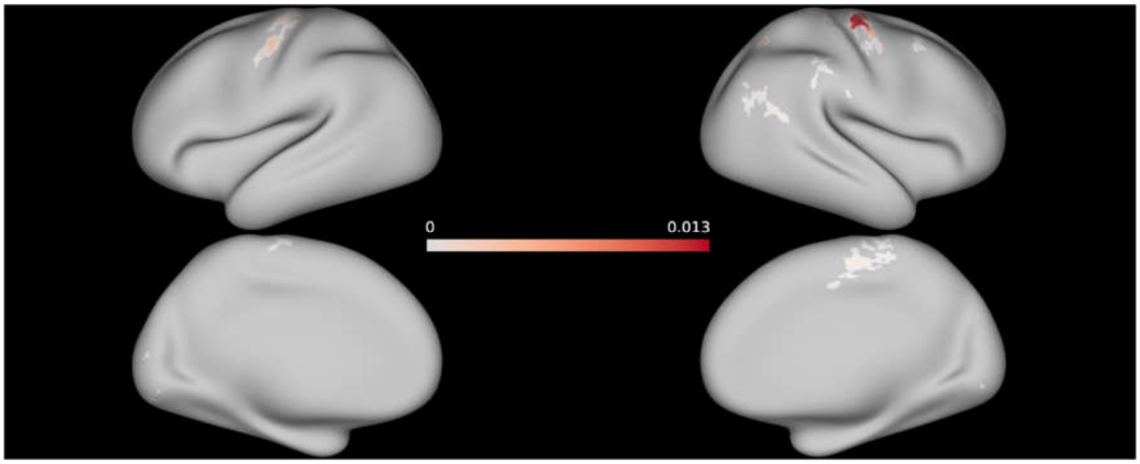

Supplementary Figure S41

Unthresholded, FWE-corrected (across contrasts, vertices, and hemispheres) t-statistical map representing areas in which performance on the Dimensional Change Card Sort Test was negatively associated with T1w/T2w ratio (N=953) when scanner site, age, sex, and SES were included as covariates of no interest.

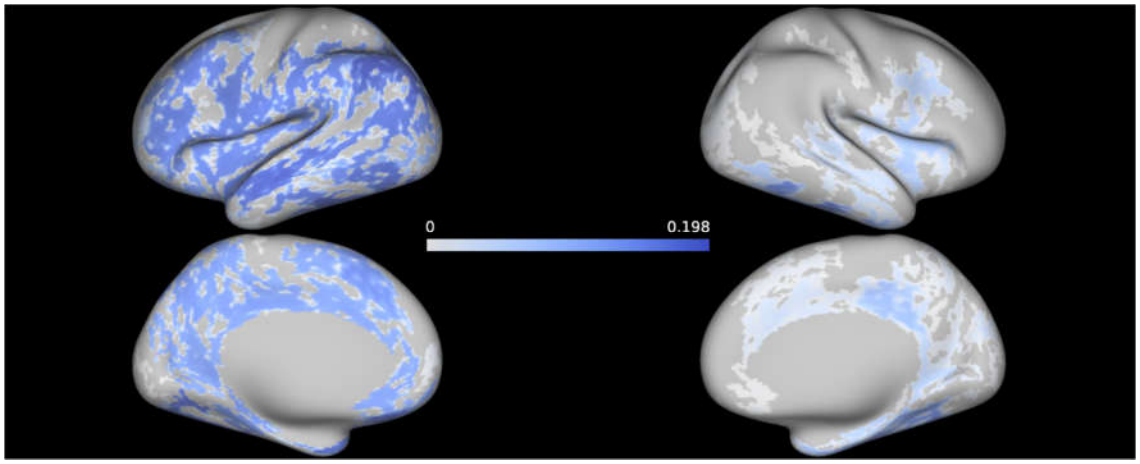

Supplement: Supplementary file 1 [file brainsci-12-00599-s001.zip › brainsci-1678776-supplementary.pdf]
